# Antibody and Cell-Mediated Immune Responses Are Correlates of Protection against Influenza Infection in Vaccinated Older Adults

**DOI:** 10.3390/vaccines9010025

**Published:** 2021-01-07

**Authors:** Chris P. Verschoor, Melissa K. Andrew, Mark Loeb, Graham Pawelec, Laura Haynes, George A. Kuchel, Janet E. McElhaney

**Affiliations:** 1 Health Sciences North Research Institute, Sudbury, ON P3E 5J1, Canada; graham.pawelec@uni-tuebingen.de (G.P.); jmcelhaney@hsnri.ca (J.E.M.); 2 Northern Ontario School of Medicine, Sudbury, ON P3E 2C6, Canada; 3 Department of Medicine (Geriatrics), Dalhousie University, Halifax, NS B3H 2E1, Canada; Melissa.Andrew@nshealth.ca; 4 Department of Pathology and Molecular Medicine, McMaster University, Hamilton, ON L8S 4L8, Canada; loebm@mcmaster.ca; 5 Department of Immunology, University of Tübingen, 72074 Tübingen, Germany; 6 UConn Center on Aging, University of Connecticut School of Medicine, Farmington, CT 06030, USA; lhaynes@uchc.edu (L.H.); kuchel@uchc.edu (G.A.K.)

**Keywords:** influenza, vaccination, older adults, correlates of protection, antibody, cell-mediated immunity

## Abstract

Despite efforts to design better vaccines for older adults, the risk for serious complications of influenza remains disproportionately high. Identifying correlates of vaccine effectiveness and understanding the heterogeneity of health outcomes in older adults are key to the vaccine development pipeline. We sought correlates of protection against laboratory-confirmed influenza illness (LCII) in a 4-year randomized trial of standard versus high-dose influenza vaccination of adults 65 years and older. To this end, we quantified serum hemagglutination-inhibition (HAI) titers and interferon-gamma (IFNγ) and interleukin-10 (IL-10) secretion by virus-challenged peripheral blood mononuclear cells. Of the 608 participants included, 26 developed either A/H3N2-(*n* = 17) or B-LCII (*n* = 9) at 10–20 weeks post-vaccination. Antibody titres for A/H3N2 at 4-weeks post-vaccination were significantly associated with protection against LCII, where every 1-standard deviation increase reduced the odds of A/H3N2-LCII by 53%. Although B-titres did not correlate with protection against B-LCII, the fold-increase in IFNγ:IL-10 ratios from pre- to 4-weeks post-vaccination was significantly associated with protection against B-LCII, where every 1-standard deviation increase reduced the odds by 71%. Our results suggest that both antibody and cell-mediated immune measures are valuable and potentially complementary correlates of protection against LCII in vaccinated older adults, although this may depend on the viral type causing infection.

## 1. Introduction

Influenza remains an important public health concern, contributing significantly to mortality, morbidity and hospitalization rates worldwide, regardless of age or socioeconomic status [1]. Moreover, older adults are disproportionately burdened by influenza [1], and in general, vaccination provides much less protection than in younger age groups. Thus, 90% of influenza deaths and most influenza hospitalizations are in older adults, and this demographic is particularly susceptible to severe outcomes of influenza A/H3N2 infection [2,3]. This is especially true when A/H3N2 is the predominant circulating strain, as hospitalization rates for adults 65 and older increase dramatically [4]. Efforts to improve influenza vaccine effectiveness in older adults have resulted in some success, such as the development of a high-dose seasonal trivalent split-virus influenza vaccine (HD-SVV), which is 24% more effective than standard dose formulation (SD-SVV) in preventing laboratory-confirmed influenza illness (LCII) in adults 65 and over, largely against A/H3N2 LCII [5]. As we have shown, HD-SVV induces significantly higher antibody responses than SD-SVV in both frail and non-frail older adults [6].

Considering the high number of participants required to power vaccine efficacy trials that are based on LCII and LCII-related outcomes, many have argued that accurate immunological correlates of protection should be given consideration as an alternative primary outcome, at least in the early phases of vaccine development [7]. This approach is especially practical for important subgroups of the population, such as older adults living with frailty and/or chronic conditions, who are at an increased risk of influenza hospitalization [8] and severity of related outcomes [9], regardless of vaccination status [10]. That being said, few correlates have been validated in older adults, and those that have are based primarily on hemagglutination inhibition antibody responses [11,12], which do not provide appreciable cross-strain immunity within the subtypes of influenza. Immunological measures such as broadly neutralizing antibodies [13] and interferon gamma (IFNγ) producing T-cells [14,15,16,17,18] are cross-reactive within the subtypes of influenza A, which may provide protection in the years when there is a vaccine strain mismatch to circulating strain of influenza virus. This has been a common occurrence with A/H3N2 strains [19,20]. Our previous studies have shown that a high ratio of IFNγ to interleukin-10 (IL-10) production in response to ex vivo influenza A/H3N2 challenge of PBMCs is a correlate of protection in older adults [21,22].

In the following study, we sought to examine both antibody- and cell-mediated immune responses as correlates of protection against influenza infection in a cohort of older adults participating in a 4-year clinical trial to compare the immunogenicity of SD-SVV versus HD-SVV seasonal vaccines. Hemagglutination-inhibition (HAI) titres and IFNγ and IL-10 responses following ex vivo influenza A/H3N2- or B-challenge were measured pre- and post-vaccination and compared between participants who developed LCII and those who did not. 

## 2. Materials and Methods

The current study was a secondary analysis of a randomized controlled trial to compare the immunogenicity and effectiveness of a high dose versus standard dose formulation of the trivalent split-virus influenza vaccine (Fluzone, Sanofi Pasteur, Lyon, France) in community-dwelling older adults (ClinicalTrials.gov: NCT02297542); the design and protocol have been previously published [6]. All participants from this trial were included, however, certain CMI measures were only performed in a subset of participants (see below). Briefly, a double-blind, re-randomization design (i.e., participants enrolled in previous years were eligible for enrollment in subsequent years) was used to measure pre-vaccination and 4-, 10- and 20-weeks post-vaccination antibody titres and cell-mediated immune parameters over four influenza seasons (October 2014–April 2015, October 2015–April 2016, October 2016–April 2017, and October 2017–April 2018). To this end, a pool of 246 unique participants (including newly recruited participants after year 1) were re-enrolled and re-randomized to standard or high dose vaccine each season (for years 1–4: 106, 175, 174 and 157, respectively) for a total of 612 study participants over the four seasons. Not all participants took part in the trial every season, but were allowed to re-enroll in subsequent years. Those who developed laboratory-confirmed influenza illness (LCII) were not excluded from the trial in subsequent years. The study protocol was approved by the Institutional Review Board of the University of Connecticut Health Centre (UCHC) and the Health Sciences North Research Ethics Board (Sudbury, ON, Canada) and all study participants provided written informed consent to participate in the study. 

### 2.1. Sites and Study Participants

Adults aged 65 years and older were recruited at the University of Connecticut Health Centre (UCHC) through the University of Connecticut Center on Aging Recruitment Core from the communities belonging to and surrounding Hartford, Connecticut, USA, and at the Health Sciences North Research Institute (HSNRI) from the community of Greater Sudbury, Ontario, Canada. Inclusion criteria included: willing to receive the influenza vaccine in the current season, at least 65 years old and vaccination in the previous influenza season. Exclusion criteria included: known immunosuppressive disorders or medications including prednisone in doses >10 mg/day, or a previous severe reaction to the vaccine due to egg, latex, or thimerosol allergies. Research coordinators ensured that vaccinations were scheduled at least 2 weeks after any acute respiratory illness.

Following informed consent, study participants were characterized according to demographic data (age, sex, ethnicity, and body-mass index (BMI)), chronic medical conditions, including known risk factors for influenza illness (pulmonary, cardiac, metabolic, renal, or neoplastic disorders), health attitudes, symptoms, and functional impairments. In order to understand and account for heterogeneity in clinical health status among the older adult participants, a Frailty Index (FI) was calculated based on 40 items validated in outcomes of influenza [23,24,25], and has been previously employed in this trial [6]. The FI counts the proportion of health deficits an individual has relative to the total number considered (in this case, 40). In general, the FI allows for the categorization of people as robust (FI < 0.10), pre-frail (0.1 < FI < 0.21), frail (0.21 ≤ FI < 0.45), and severely frail (FI ≥ 0.45) [26]. Blood samples were collected at the pre-vaccination and 4-, 10- and 20-week post-vaccination visits. Given its known relationship with vaccine responsiveness and susceptibility to influenza infection [18,27], cytomegalovirus (CMV) serostatus was determined in serum using a CMV IgG ELISA kit (Genesis Diagnostics Inc., Cambridgeshire, UK) according to the manufacturer’s instructions.

### 2.2. Influenza Vaccination and Surveillance

Participants were randomized 1:1 to receive either standard or high dose vaccine (Fluzone, Sanofi Pasteur) at each study site, and vaccines were administered by a research nurse not associated with the study. Clinical and laboratory research staff remained blinded to the vaccine group until all laboratory assays were completed.

Influenza surveillance included weekly contact with study subjects to assess flu-like symptoms or acute respiratory infection (ARI); this included upper (coryza or sore throat) or lower (cough or shortness of breath) respiratory tract symptoms, headache, malaise, myalgia, or fever (>99 °F or 37.3 °C orally or 100 °F rectally) [28]. Upon documentation of an ARI, nasopharyngeal swabs were collected (within 5 days of onset of symptoms) for polymerase chain reaction (PCR) detection of influenza virus. A post-influenza season (i.e., 20 weeks post-vaccination) blood draw was also obtained from all participants in order to detect an antibody response to infection over the influenza season (see below). Routine screening for symptoms of ARI also occurred at the 4-, 10- and 20-week visits when blood samples were collected. Influenza illness (i.e., LCII) was documented by PCR detection of influenza virus following an ARI or evidence of seroconversion (4-fold rise in antibody titres; see below for the specific antigens employed to detect seroconversion in each season) at 20-weeks post-vaccination. In total, 30 participants were diagnosed with LCII throughout the 4-year study.

### 2.3. Antibody and Cell-Mediated Immune Response Measures

Hemagglutination-inhibition (HAI) antibody titres were quantified using previously described standard methods [29,30]. Influenza types used for HAI testing were as follows: Year 1, A/Texas/50/2012 and B/Massachusetts/2/2012; Year 2, A/Switzerland/9715292-2013 and B/Phuket/3073/2013; Year 3, A/Hong Kong/4801-2014 and B/Brisbane/60/2008; and Year 4, A/HongKong/4801/2014 and B/Brisbane/60/2008. Laboratory testing was conducted after each study year, and participant serum was randomized before plating.

Cell-mediated immune (CMI) measures were assessed using previously described standard operating procedures [31] and validated according to the International Council for Harmonisation of Technical Requirements for Pharmaceuticals for Human Use [32]. Briefly, thawed PBMCs were stimulated with sucrose-gradient purified, live influenza virus (A/Victoria/3/75 or B/Lee/40; Charles River, MA, USA) at a multiplicity of infection of 2 in AIM V media (Life Technologies, Burlington, Canada) and incubated at 37 °C/5% CO_2_ for 20 h. These influenza virus strains were chosen for consistency across multi-year studies and contain hemagglutinin and internal protein (matrix 1 and nucleoprotein) peptide sequences that are shared and thus cross-reactive across all A/H3N2 or B strains. Supernatants and lysates were collected and stored at −80 °C until assay measurement. Concentrations of IFNγ and IL-10 were measured in supernatants by multiplexed bead ELISA (Millipore, Toronto, Canada) and reported as pg/mL. Laboratory testing for cytokine concentration was performed after each study year, except for IFNγ and IL-10 in supernatants from influenza B stimulated cultures. This was conducted after all testing had been completed, and only on participants who developed LCII and a matched subset of participants who did not. Matching of non-LCII and LCII participants was performed with the R package “MatchIt” using a propensity score based approach; briefly, propensity scores were estimated by logistic regression, adjusting for age, sex, frailty, BMI, site, year, vaccine dose and CMV serostatus, and matched at a ratio of 3 non-LCII to 1 LCII using a nearest-neighbor approach. Of the 90 non-LCII participants identified from this process, 80 were randomly selected so that the final subset of participants was 110 (i.e., 30 LCII and 80 non-LCII). Group-wise comparisons indicated no significant differences in any of the aforementioned factors used to generate propensity scores between LCII and non-LCII participants in the matched subset. Furthermore, associations between CMI measures from A/H3N2-stimulated cultures and A/H3N2-LCII (as described below) were comparable to that of the entire cohort (data not shown).

### 2.4. Statistical Analysis

Participant demographics (i.e., age, sex, dose, site, CMV serostatus, BMI, and frailty) were summarized as the mean and standard deviation or count and frequency, and group-wise comparisons performed by *t*-test or chi-square test, respectively; HAI and CMI were summarized as the geometric mean and 95% confidence interval. Group-wise comparisons between LCII and non-LCII participants were performed on the entire cohort, as well as within frailty categories. Non-LCII participant demographics were previously reported by our research group [6].

The primary goal of the current study was to correlate HAI and CMI measures at baseline and 4-weeks post-vaccination with A/H3N2- or B-LCII at 10- to 20-weeks post-vaccination. Logistic regression was performed, where each model included the fixed effect of a single HAI/CMI measure and was adjusted for year (i.e., flu season). For models where A/H3N2-LCII was the endpoint, year was included as a random intercept, where for B-LCII, it was included as a fixed effect since the intraclass correlation was nearly zero, but nonetheless improved model fitness. For each HAI/CMI measure that was found to be significantly associated with LCII, a sensitivity analysis was performed where additional models including the fixed effect of a single participant demographic variable were tested to ensure robustness of the association. Participant demographics were also associated to LCII using the aforementioned mixed logistic model. Odds ratios (OR) and 95% confidence intervals were reported and estimates were considered statistically significant if the confidence interval did not cross an OR of 1. To ensure comparability of estimates for HAI/CMI measures, natural log-transformed values were standardized so that every unit represented a 1-standard deviation change; hence, ORs represent the likelihood of A/H3N2- or B-LCII per 1-standard deviation change in any given measure. All analyses were conducted in the R environment.

## 3. Results

### 3.1. Characteristics of LCII and Non-LCII Participants

Over the course of our 4-year trial, 30 of 612 participants developed LCII (HD group, *n* = 10; SD group, *n* = 20). Due to the small number of cases, participants that developed A/H1N1-LCII (*n* = 3) were removed from further analysis, and one A/H3N2 case was removed due to missing laboratory data. Hence, the final sample of participants employed in our study was 608, 26 of whom developed LCII (A/H3N2, *n* = 17; B, *n* = 9); of these 26, 16 were confirmed by both serology and PCR (A/H3N2, *n* = 8; B, *n* = 8), and the remaining by serology only (Table 1). The majority of these cases occurred in year 4 (2017–2018, *n* = 15), with the next largest number in year 1 (*n* = 7), then year 2 (*n* = 3) and only one in year 3. The characteristics of those participants who developed LCII were nearly identical to those that did not with respect to age (total mean ± standard deviation: 76 ± 7.4), BMI (28 ± 4.8), Frailty Index (0.11 ± 0.07) and CMV serostatus (frequency of negatives: 47%) (Table 2). There was no difference in the proportion of LCII and non-LCII participants when stratified by frailty level (data not shown). Relative to participants who did not develop LCII, cases were more likely to be male (50% vs. 32%), have received the standard dose vaccine (65% vs. 51%), and be enrolled at the HSNRI site (73% vs. 58%), although none of these differences were statistically significant.

### 3.2. Changes in HAI and CMI Measures over Time in LCII and Non-LCII Participants

**Figure 1:**
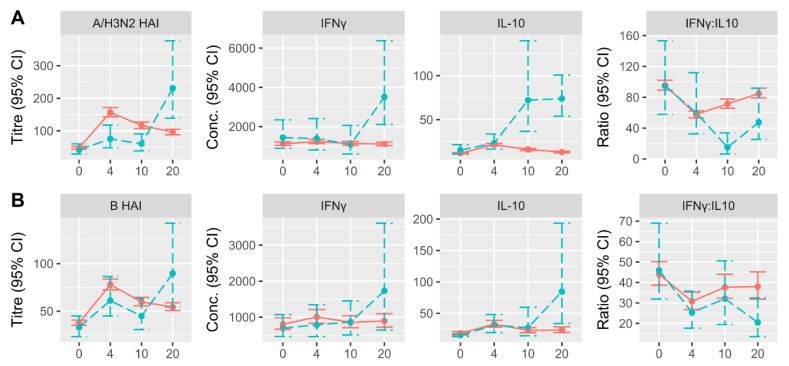
Change in antibody (HAI) titres and cell-mediated immune (CMI) measures over time depending on LCII-status at 10–20 weeks post-vaccination. Participants who developed (**A**) A/H3N2-LCII (*n* = 17) and (**B**) B-LCII (*n* = 9) are shown as blue, dashed lines, whereas participants who did not develop LCII (*n* = 582) are presented as red, solid lines. Measures at baseline (0) and 4-, 10- and 20-weeks post-vaccination are presented as the geometric mean and 95% confidence interval, of which, HAI titres are strain-specific, and CMI measures are in response to ex vivo challenge with either influenza A/H3N2 (**A**) or B (**B**). Note: IFNγ and IL-10 responses to B challenge were only measured in a subset of 80 non-LCII participants. Conc., concentration.

Antibody titres uniformly peaked at 4-weeks post-vaccination and declined thereafter, with the exception of participants who developed LCII, as seroconversion at 20-weeks was evident (Figure 1; Supplemental Table S1). Prior to 20-weeks, A/H3N2 HAI titres in participants that developed A/H3N2-LCII, and influenza B titres in participants who subsequently developed B-LCII tended to be lower at all time points. Interferon gamma and IL-10 measures tended to peak at 4 weeks post-vaccination, and there was a significant decrease in the IFNγ:IL-10 ratio (Figure 1). Participants who developed LCII exhibited an increase in cytokine concentrations and a further decrease in the IFNγ:IL-10 ratio at 10- and/or 20-weeks depending on the timing of the influenza season in each study year, and the response patterns of non-LCII participants to A/H3N2- or B-challenge were nearly identical (Figure 1). To note, the patterns of antibody responses by non-LCII participants have been published previously by our group [6].

### 3.3. Associations of Participant Factors with the Likelihood of LCII

**Figure 2:**
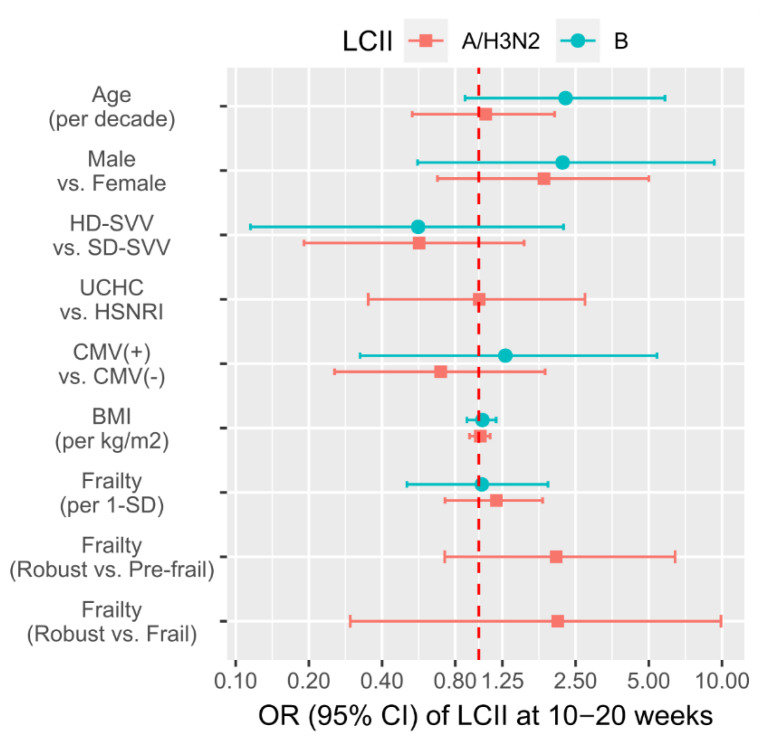
The association of participant demographics with the likelihood of laboratory-confirmed influenza illness (LCII). The odds ratio (OR) and 95% CI of LCII at 10–20 weeks post-vaccination are presented for age, sex, dose, site, CMV serostatus, BMI, and frailty. Estimates are relative to non-LCII participants (*n* = 582) and were derived separately for A/H3N2-LCII (*n* = 17; square points) and B-LCII (*n* = 9; round points) cases. Those estimates above the red dotted line (no difference) indicate a greater likelihood of LCII as compared to the reference group (i.e., second listed value), or per the relative unit as described in brackets. Note: since no B-LCII cases occurred at UCHC or in participants categorized as frail, estimates for site of frailty as a categorical variable could not be derived.

To determine whether demographic factors or HAI/CMI measures were predictive of LCII at 10–20 weeks post-vaccination, we performed logistic regression. Although the estimated odds of either A/H3N2- or B-LCII were notably increased with older age, male sex, SD-SVV vaccination and frail or pre-frail relative to robust frailty status, none of these associations were significant (Figure 2).

**Figure 3:**
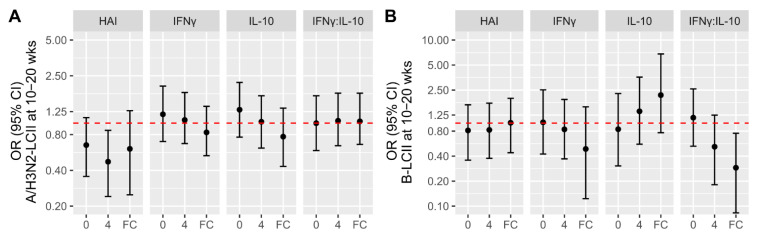
The association of antibody (HAI) titres and cell-mediated immune (CMI) measures with the likelihood of laboratory-confirmed influenza illness (LCII). The odds ratio (OR) and 95% CI of LCII at 10–20 weeks post-vaccination are presented. Estimates are relative to non-LCII participants (*n* = 582) and were derived for measures at baseline (0), 4-weeks, and the fold-change (FC) difference between the two time points, separately for (**A**) A/H3N2-LCII (*n* = 17) and (**B**) B-LCII (*n* = 9) cases. HAI titres are for A/H3N2 (**A**) and B (**B**) types, and CMI measures are in response to ex vivo challenge with either influenza A/H3N2 (**A**) or B (**B**). Those estimates above the red dotted line (no difference) indicate a greater likelihood of LCII for every 1-standard deviation change in the natural log-transformed measure.

Antibody titres at baseline and 4-weeks post-vaccination were found to be associated with greater protection against LCII, although significance varied by influenza type and time point (Figure 3). Of note, for every 1-standard deviation increase in A/H3N2 titres at 4-weeks post-vaccination, the odds of A/H3N2-LCII decreased by 53% (OR (95% CI]): 0.47 (0.24, 0.87)); the estimated odds of A/H3N2-LCII per 1-standard deviation increase in the A/H3N2 titre fold-change from pre- to 4-weeks post-vaccination also decreased nearly 40%, but was not statistically significant (0.61 (0.25, 1.27)) (Figure 3A). When only PCR-confirmed A/H3N2-LCII cases were included in the analysis (*n* = 8), the odds of LCII relative to 4-week post-vaccination A/H3N2 titres was similar, but also not significant (0.498 (0.19, 1.15)). Increasing B HAI titres were weakly associated with protection against B-LCII, and did not reach statistical significance (Figure 3B).

With respect to cell-mediated immune measures, neither the IFNγ nor the IL-10 response to A/H3N2-challenge, nor the IFNγ:IL-10 ratio, were associated with the likelihood of A/H3N2-LCII (Figure 3A). However, for every 1-standard deviation fold-increase in the IFNγ:IL-10 ratio (following ex vivo influenza B challenge) from pre- to 4-weeks post-vaccination, the odds of B-LCII decreased by 71% (OR [95% CI]: 0.29 [0.08, 0.76])(Figure 3B); when only PCR-confirmed B-LCII cases were included in the analysis (*n* = 8), this estimate was very similar (0.186 (0.04, 0.58)).

## 4. Discussion

Immunological correlates of protection are powerful tools to guide the development of improved vaccines against influenza and other acute infections, and to better understand the underlying biological context of vaccine effectiveness. While a few correlates of protection against influenza have been established, most are related to the host antibody response and are poorly described in older adults, arguably the largest and most vulnerable demographic with regards to vaccine failure and overall susceptibility to infection [33]. Hence, we sought to perform a secondary analysis of our recent randomized trial comparing the HD and SD influenza vaccine in order to investigate both antibody and cell-mediated immune correlates of protection in older adults.

In this analysis, only cases of A/H3N2- and B-LCII were included, the majority of which occurred in years 1 (2014/15) and 4 (2017/18). A major vaccine strain mismatch occurred in both years, during which A/H3N2 strains predominated (as we observed). In Year 1, A/Texas/50/2012 (A/H3N2) was the vaccine strain and A/Switzerland/9715292-2013 was the circulating virus and overall vaccine effectiveness was less than 10% in the United States [34]. In Year 4, A/Hong Kong/4801/2014 (A/H3N2) was the vaccine strain and the predominant circulating virus A/Washington/16/2017, and vaccine effectiveness was 17% in Canada [35]. Despite this mismatch, A/H3N2 HAI titres 4-weeks post-vaccination were significantly associated with protection against A/H3N2-LCII. Although point estimates suggest that influenza B titres pre- and post-vaccination also reduce the odds of B-LCII by approximately 20%, neither was statistically significant. This may have been due to the relatively lower number of B-LCII cases that occurred (*n* = 9), 3 of which were during the 2015/16 season when vaccine effectiveness in adults 65 and older against influenza B was particularly low (i.e., −34% [36]), and the remaining cases were in 2017/18 when the vaccine (Victoria) and circulating (Yamagata) strains were from different influenza B lineages [35]. For both A/H3N2- and B-LCII, associations with respective antibody titres were very similar when only PCR-positive LCII cases were considered.

Although we found increased A/H3N2 HAI titres to be associated with reduced risk of LCII, neither the secretion of IFNγ or IL-10 by participant PBMCs following ex vivo A/H3N2-challenge was associated with the risk of A/H3N2-LCII. However, we did find that the IFNγ:IL-10 ratio following ex vivo influenza B challenge was significantly associated with a reduction in the risk of B-LCII; specifically, an increase in the ratio from baseline to 4-weeks post-vaccination afforded greater protection against B-LCII. To the best of our knowledge, our findings regarding B-LCII have not been previously reported, while our findings regarding A/H3N2-LCII differ from our previous work. In one study we showed that increasing IFNγ:IL-10 ratios at pre-and 4-weeks post-vaccination were associated with protection against A/H3N2-LCII during the 2003–2004 influenza season when there was an A/H3N2 vaccine strain mismatch and influenza began to circulate in late November [37]. In another study when there was a good match to the circulating strain and influenza did not begin to circulate until after the 10-week post-vaccination time point, we found that a low IFNγ:IL-10 ratio at pre- and 4-weeks post-vaccination predicted increased risk for A/H3N2-LCII only in those who were PCR+ and had febrile (more severe) LCII; importantly this group did not seroconvert to influenza infection [21]. Thus, there are distinct differences that may explain the discrepancy in the findings from these studies compared to the current one: in the former study, lower IFNγ:IL-10 ratios in LCII cases may reflect competing responses to vaccination and to influenza virus when infection occurs within 4 weeks of vaccination [37]; in the latter study, the vaccine was well matched to the circulating A/H3N2 strain and influenza infection did not occur until after the 10-week post-vaccination time point [21]. The current study differed from both of our previous studies in that the vaccine A/H3N2 strain was not well matched to the circulating strain, and influenza began to circulate before the 10-week post-vaccination time point in the 2013–2014 and 2017–2018 seasons where most of the A/H3N2 cases were identified. Furthermore, we did not stratify our participants according to the severity of LCII that they developed.

Our study had notable strengths, and some limitations. First, ours is among the very few studies that investigated both antibody and cell-mediated immunity-based assays as correlates of protection in older adults. Second, our study design spanned multiple influenza seasons at two geographically distinct sites and included the collection of data pertaining to multiple determinants of health and immunity in older adults. Importantly, our collective studies have demonstrated the importance of vaccine strain match to the circulating strain and timing of the influenza season relative to vaccination, in establishing correlates of protection. Limitations of the study include a relatively small number of LCII cases, which reduced statistical power, and our reliance on serology or PCR-detection of virus in order to confirm LCII in participants, which may have led to misspecification [38]. That being said, when we excluded participants that were diagnosed with LCII only by serology, our associations of post-vaccination A/H3N2 antibody titres or the fold-change in IFNγ:IL-10 with LCII changed very little.

## 5. Conclusions

In summary, we have shown that in older adults randomized to either SD-SVV or HD-SVV HAI, antibody titres are a significant correlate of protection against A/H3N2-LCII, while the pre- to post-vaccination IFNγ:IL-10 ratio is a significant correlate of protection against B-LCII. Hence, based on current and previous work, the identification of correlates of protection may depend on the following factors: specificity of the response to virus challenge, similarity of the vaccine strain to the circulating strain, the influenza type (A vs. B) and subtype (A/H3N2 vs. A/H1N1), and timing of the influenza season relative to the time of vaccination for each of the vaccine strains. These findings contribute to a small, yet growing body of evidence pertaining to the immunological factors that predict influenza risk in older adults. Further work to confirm the roles of neutralizing antibodies (e.g., HAI or anti-NA titres) and CMI measures such as IFNγ and IL-10 secretion is warranted, in addition to studies exploring the potential roles of novel and understudied immune parameters as correlates of protection in older adults.

## Figures and Tables

**Table 1:** Summary of laboratory-confirmed influenza illness (LCII) diagnoses during the study.

	A/H3N2-LCII			B-LCII			Negative
	Either	HAI Only	HAI + PCR	Either	HAI Only	HAI + PCR	Both
All years	17	9	8	9	1	8	582
2014/2015	7	5	2	0	0	0	99
2015/2016	0	0	0	3	1	2	169
2016/2017	1	1	0	0	0	0	173
2017/2018	9	3	6	6	0	6	141

Count of LCII cases, diagnosed by either HAI (i.e., seroconversion) or PCR assay, HAI only or both assays, presented for A/H3N2 and B influenza. Negative, participants not diagnosed with LCII by either method.

**Table 2:** Demographics of participants enrolled, subdivided according to LCII status.

		LCII at 10–20 Weeks		
	Total	Negative	Positive	*p*-Value
	(*n* = 608)	(*n* = 582)	(*n* = 26)	
**Age**	76 (7.38)	76 (7.41)	77 (6.68)	0.50
Missing	1 (0.2%)	1 (0.2%)	0 (0%)	
**Sex**				0.09
Female	408 (67.1%)	395 (67.9%)	13 (50.0%)	
Male	200 (32.9%)	187 (32.1%)	13 (50.0%)	
**Dose**				0.21
SD	313 (51.5%)	296 (50.9%)	17 (65.4%)	
HD	295 (48.5%)	286 (49.1%)	9 (34.6%)	
**Site**				0.17
HSNRI	354 (58.2%)	335 (57.6%)	19 (73.1%)	
UCHC	254 (41.8%)	247 (42.4%)	7 (26.9%)	
**CMV Status**				0.89
Negative	284 (46.7%)	271 (46.6%)	13 (50.0%)	
Positive	324 (53.3%)	311 (53.4%)	13 (50.0%)	
**BMI**	28 (4.84)	28 (4.84)	29 (4.68)	0.33
Missing	3 (0.5%)	3 (0.5%)	0 (0%)	
**Frailty Index (continuous)**	0.11 (0.0735)	0.11 (0.0736)	0.12 (0.07)	0.57
Missing	2 (0.3%)	2 (0.3%)	0 (0%)	
**Frailty Index (categorical)**				0.40
Robust	303 (49.8%)	293 (50.3%)	10 (38.5%)	
Pre-frail	249 (41.0%)	235 (40.4%)	14 (53.8%)	
Frail	54 (8.9%)	52 (8.9%)	2 (7.7%)	
Missing	2 (0.3%)	2 (0.3%)	0 (0%)	

Continuous factors are summarized as mean (standard deviation) and categorical factors as count (frequency), and statistical significance (*p*-value) was determined by t-test and chi-square test, respectively.

## Data Availability

The data presented in this study are available on reasonable request from the corresponding author. The data are not publicly available due to ethical restrictions.

## Supplementary Materials

The following are available online at https://www.mdpi.com/2076-393X/9/1/25/s1, Table S1: Summary of antibody and cell-mediated immune response measures for participants at each time point, subdivided according to LCII status.
