# Supplementary material for: Antibody and Cell-Mediated Immune Responses Are Correlates of Protection against Influenza Infection in Vaccinated Older Adults
Source: Vaccines (Basel). 2021 Jan 7;9(1):25. doi: 10.3390/vaccines9010025 (PMC7825602; doi:10.3390/vaccines9010025)
Supplement: Supplementary file 1 [file vaccines-09-00025-s001.pdf]

Supplemental Table S1: Summary of antibody and cell-mediated immune response measures for participants at each time point, subdivided according to LCII status.

			LCII at 10-20 weeks	
		**Negative (N=582)**	**A/H3N2 (N=17)**	**B (N=9)**
**A/H3N2 HAI**	**Baseline**	47.9 (44, 52.4) [4]	41.7 (28.9, 60.1) [0]	48.5 (26.2, 86.4) [0]
	**4**	156 (141, 173) [6]	75.3 (51.1, 120) [0]	154 (63.5, 388) [0]
	**10**	116 (106, 128) [19]	60.1 (40, 90.4) [0]	109 (40, 296) [0]
	**20**	96.2 (87.3, 106) [22]	231 (139, 369) [0]	109 (46.7, 296) [0]
**B HAI**	**Baseline**	37.8 (35.3, 40.3) [4]	46.1 (32, 70.8) [0]	33 (23.3, 46.7) [0]
	**4**	78 (72.1, 83.6) [6]	72.2 (51.1, 116) [0]	61.1 (44.9, 86.4) [0]
	**10**	59.7 (55.5, 63.9) [19]	56.6 (38.4, 86.8) [0]	44.9 (30.5, 63.5) [0]
	**20**	54.3 (50.4, 58.1) [22]	50.1 (34.7, 78.4) [0]	89.8 (56.5, 148) [0]
**IFNγ (A/H3N2)**	**Baseline**	1120 (1030, 1220) [6]	1440 (848, 2460) [0]	2610 (1770, 3730) [0]
	**4**	1240 (1140, 1340) [8]	1390 (762, 2400) [0]	2800 (1830, 4120) [0]
	**10**	1140 (1030, 1260) [16]	1080 (623, 2040) [0]	3000 (1770, 4860) [0]
	**20**	1110 (1020, 1210) [29]	3510 (2010, 5940) [1]	2770 (1390, 5050) [0]
**IL-10 (A/H3N2)**	**Baseline**	11.7 (11.1, 12.4) [5]	15.2 (10.7, 21.6) [0]	16.8 (14.2, 19.8) [0]
	**4**	21.4 (20.2, 22.9) [8]	23.2 (16.3, 33.8) [0]	28.8 (23, 35.6) [0]
	**10**	16 (14.9, 17.3) [19]	72.3 (36.5, 139) [0]	19.1 (15.1, 23.6) [0]
	**20**	13.1 (12.3, 14) [28]	73.9 (53.9, 103) [1]	15.9 (10.7, 21.7) [0]
**IFNγ (B)**	**Baseline**	803 (670, 966) [503]	1120 (747, 1760) [0]	694 (455, 1040) [0]
	**4**	1010 (831, 1220) [503]	1270 (917, 1680) [0]	789 (473, 1330) [0]
	**10**	859 (706, 1040) [504]	903 (554, 1400) [1]	865 (514, 1430) [0]
	**20**	894 (737, 1100) [507]	1190 (857, 1710) [1]	1730 (722, 3690) [0]
**IL-10 (B)**	**Baseline**	18.2 (15.5, 21.3) [503]	28.3 (21.2, 40.1) [0]	15.1 (13.1, 17.6) [0]
	**4**	32.7 (27.7, 38.7) [503]	45.7 (33, 65) [0]	31.1 (19, 49.6) [0]
	**10**	22.8 (19.1, 27.1) [504]	46.4 (28.8, 72.3) [1]	27 (14.4, 60.2) [0]
	**20**	23.5 (19.5, 28.5) [507]	31.3 (23.3, 43.4) [1]	84.5 (33, 203) [0]

Hemagglutination inhibition (HAI) was measured for the seasonal A/H3N2 and B types at baseline (pre-vaccination) and 4-, 10- and 20-weeks post-vaccination. Cell-mediated immune measures IFNγ and IL-10 were measured at the same time points, in response to *ex vivo* challenge with either A/H3N2 or B types (in brackets). All measures are presented as the geometric mean (95% confidence interval), and the count of missing data in square brackets. * IFNγ and IL-10 responses to influenza B were measured in a subset of 110 participants. LCII, laboratory-confirmed influenza illness.
